# *Heteropterys rosmarinifolia*, a new species of Malpighiaceae with verticillate leaves from savannas grasslands of central Brazil

**DOI:** 10.3897/phytokeys.175.62953

**Published:** 2021-03-19

**Authors:** Rafael F. Almeida, Marco O. O. Pellegrini

**Affiliations:** 1 Independent researchers, CEP 25651-090, Petrópolis, Rio de Janeiro, Brazil Unaffiliated Rio de Janeiro Brazil

**Keywords:** Cerrado, Malpighiales, Neotropical flora, Tetrapteroid clade, xeric plants

## Abstract

A new species of *Heteropterys* belonging to the Parabanisteria informal group is described for the savannas grasslands of the Serra do Tombador Natural Reserve, municipality of Cavalcante, State of Goiás, Brazil. *Heteropterys rosmarinifolia* is easily distinguished from the remaining Brazilian species of the Parabanisteria informal group due to its stems unbranched, leaves associated with the inflorescence verticillate, blades linear to very narrowly oblong, strongly conduplicate, ascending to upright, and sparsely sericeous to glabrous at age. We present a complete morphological description for the new species, photographic plates, comments on its distribution, ecology, and taxonomy, besides an identification key to the species of *Heteropterys* from the Parabanisteria group from the State of Goiás, Brazil.

## Introduction

*Heteropterys* Kunth is the largest genus of Neotropical Malpighiaceae, with ca. 160 species occurring in various open to closed habitats from northern Mexico to northern Argentina (Anderson et al. 2006; POWO 2021). A single species, *H. leona* (Cav.) L., is also widely and naturally distributed in western Africa (Anderson 2001). The genus includes a great deal of morphological variation in life forms (i.e., subshrubs, shrubs, lianas to small trees), gland position on the leaves (i.e., glands inserted at the base, middle or apex of petioles and/or along the leaf blade and/or on margins), inflorescence architecture (i.e., 1-flowered cincinni arranged in thyrsi, corymbs, or umbels), petal color (i.e., white, yellow, orange, pink, red to purple), petal shape when in bud (i.e., smooth or dorsally keeled), and shape of the style apex (i.e., rounded, truncate, uncinate, or hammer-shaped) (Amorim 2003; Pessoa et al. 2014; Almeida et al. 2021). The only morphological character shared by all species in the genus is the schizocarpic fruit with winged mericarps, in which the dorsal wing is more developed than the lateral ones, thickened on the inferior side, and curved towards the floral center (Almeida et al. 2021). This peculiar character is unique in Malpighiaceae and currently the sole synapomorphy of the monophyletic *Heteropterys* (Almeida & van den Berg, 2021).

The immense morphological variation in *Heteropterys* makes it one of the most taxonomically complex genera in the family, with no modern revision (Almeida pers. observ.). Nonetheless, seven informal groups, derived from different taxonomic ranks proposed in the past, are currently recognized in the literature: Apytchia, Madarophyllis, Metallophyllis, Parabanisteria, Rhodopetalis, Stenophyllarion, and Xanthopetalis (Amorim 2003). Out of those, the Parabanisteria group is the most diversified, with over 50 species confined to Neotropical savannas from the Amazon domain (i.e., campinaranas), Central Brazil and Bolivia, and the llanos from Colombia and Venezuela (POWO 2021; Almeida pers. observ.). Species of Parabanisteria are easily recognized by their large flowers arranged in thyrsi and covered in brown hairs, the apex of sepals revolute at anthesis, petals bright yellow and usually widely elliptic, and lateral wings of mericarps usually absent (Almeida et al. 2021).

During the preparation of the *Heteropterys* monograph for the Flora do Brasil 2020 project (Almeida et al. 2021), we came across two recently collected specimens of *Heteropterys* belonging to the Parabanisteria group with unusual verticillate leaves from Serra do Tombador, municipality of Cavalcante, State of Goiás. We describe these specimens as a new species, presenting detailed morphological descriptions, photo plates, and comments on its distribution, ecology, and taxonomy.

## Methods

Morphological data were based on herbaria samples (HUEFS, RB, and UFRN; acronyms according to Thiers, continuously updated). The indumentum terminology follows Anderson (1981), structure shapes follow Radford et al. (1974), inflorescence terminology and morphology follow Weberling (1965, 1989), and fruit terminology follows Spjut (1994) and Anderson (1981). The conservation status was proposed following the recommendations of IUCN Red List Categories and Criteria, Version 3.1 (IUCN 2012). GeoCAT (Bachman et al. 2011) was used for calculating the Extent of Occurrence (EOO) and the Area of Occurrence (AOO).

## Results

### Heteropterys rosmarinifolia

Taxon classificationPlantaeMalpighialesMalpighiaceae

R.F.Almeida & M.Pell. sp. nov.

27D1D96A-3376-5AC8-9CB2-E1532A970E2A

urn:lsid:ipni.org:names:77215898-1

Figs 1 , 2 , 3

#### Diagnosis.

*Heteropterys rosmarinifolia* differs from the remaining Brazilian species of the Parabanisteria group due to its stems unbranched (vs. branched), leaves associated with the inflorescence verticillate (vs. opposite to subopposite), blades linear to very narrowly oblong (vs. several shapes, but never linear or narrowly oblong), strongly conduplicate (vs. plane), ascending to upright (vs. patent).

#### Type.

**Brazil. Goiás**: Cavalcante, Reserva Natural Serra do Tombador, road GO-241, estrada de terra para o Engenho II, a direita da estrada, 13°42'00"S, 47°48'00"W, fl., 25 Jul 2014, R. Sartin et al. 576 (holotype: UFRN barcode UFRN00024927!; isotype: RB barcode RB01408371!).

#### Description.

*Subshrubs* 25–55 cm tall, unbranched. *Xylopodium* not seen. *Branches* densely rusty-sericeous, hairs adpressed; internodes 0.9–3.6 cm long. *Stipules* 15–20 mm long, interpetiolar, fused, both sides densely rusty-sericeous. *Leaves* subopposite at base in vegetative branches, becoming opposite at mid-length, and verticillate towards the apex of the vegetative branches and in flowering branches; petioles 0.2–0.3 cm long, canaliculate, densely rusty-sericeous, eglandular; blades 1.3–8.3 × 0.1–0.3 cm, linear to very narrowly oblong, strongly conduplicate, ascending to upright, coriaceous, eglandular, base obtuse, margins entire, plane to slightly involute, apex obtuse to rounded, sparsely rusty-sericeous to glabrous, abaxially rusty-sericeous, becoming only sparsely rusty-sericeous along the midvein with age. *Synflorescence* consisting of a solitary main florescence, leaves associated with the inflorescence much reduced, sessile, 3.1–14 × 0.8–1.0 mm. *Thyrsi* 4–16-flowered, terminal, pedunculate, many-branched; main axis 1.0–2.8 cm long, smooth, densely rusty-sericeous; bracts 2.0–4.3 × 0.9–1.3 mm, elliptic, concave, patent, eglandular, adaxially glabrous, abaxially rusty-sericeous; cincinni verticillate, 3–4 per node, 1-flowered; peduncle 3.0–5.1 mm long, rusty-sericeous; bracteoles opposite, inserted at the apex of the peduncle, 2.0–2.5 × 0.5–0.9 mm, elliptic to broadly elliptic, concave, patent, adaxially glabrous, abaxially rusty-sericeous. *Flowers* 1.0–1.2 cm diam. at anthesis; floral buds 3.9–5.0 × 2.8–4.0 mm, transversally widely oblongoid to widely depressed ovoid, apex obtuse to truncate; pedicel 5.0–7.0 mm long, rusty-sericeous. *Sepals* 3.0–4.8 × 1.0–1.9 mm, straight, smooth, apex rounded, strongly revolute at anthesis, adaxially glabrous, abaxially densely rusty-sericeous; the anterior eglandular, the latero-anterior 1-glandular, the posterior 2-glandular, glands 1.0–1.2 × 0.5–0.6 mm, green, elliptic. *Petals* bright yellow, dorsally smooth in bud, persistent at anthesis; lateral petals orbicular, plane, limb 3.4–4.2 × 2.7–3.5 mm, margin denticulate, eglandular, claws 1.4–2.2 × 0.4–0.6 mm, both sides glabrous; posterior petal orbicular, erect, limb 3.1–3.5 × 3.0–3.4 mm, base truncate, margin denticulate, eglandular, claws 1.0–2.1 × 0.6–0.8 mm, both sides glabrous. *Stamens* 10, all fertile, filaments 2.2–3.0 × 0.2–0.8 mm, basally connate for 0.3–1.2 mm, cylindrical, thicker at base, tapering towards the apex; connective glandular, white, glabrous; thecae 0.8–1.0 × 0.35–0.40 mm, white, glabrous. *Ovary* 1.4–1.8 × 1.1–1.5 mm, ovoid, densely sericeous; styles 3, erect, 2.4–2.9 × 0.5–0.7 mm, cylindrical, divergent at apex, glabrous, apex rounded, anterior style slightly shorter than posterior ones; stigmas lateral, pointing towards the center of the flower. *Fruits* not seen. *Seeds* not seen.

#### Paratype.

**Brazil. Goiás**: Cavalcante, Reserva Natural Serra do Tombador, caminho para a cachoeira da Ave Maria, ponto onde se vê a cachoeira, 13°44'26"S, 46°52'46"W, 22 Sep 2015, fl., L. Rocha et al. 668 (HUEFS barcode HUEFS000273192!).

#### Distribution, habitat, and phenology.

**Figure 1.**
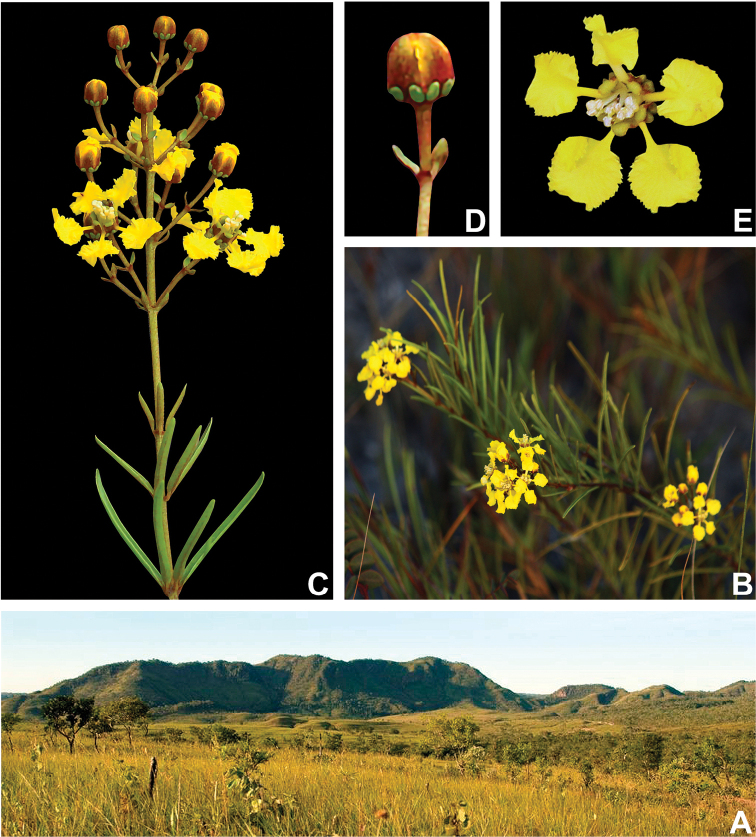
*Heteropterys rosmarinifolia* R.F.Almeida & M.Pell. **A** savannah grasslands from Reserva Natural Serra do Tombador **B** habit **C** detail of the apex of the branch, showing the verticillate leaves and solitary inflorescence **D** cincinnus, showing the concave bracteoles and the floral bud **E** front view of the flower. All photos by R. Sartin, except **A** by H. Palo-Jr.

*Heteropterys rosmarinifolia* is known only from savannas grasslands within the Serra do Tombador Natural Reserve in the State of Goiás, Brazil (Fig. 1). It blooms from July to September, but fruits are unknown.

#### Conservation status.

*Heteropterys rosmarinifolia* is known only from two collections, probably from the same population within the limits of the Serra do Tombador Natural Reserve in the State of Goiás, Brazil. Until additional fieldwork can be done in the savannas of northern Goiás, this species is best categorized as data deficient (DD).

#### Etymology.

The epithet makes reference to the leaves of the new species that resemble those of the widely cultivated aromatic herb, rosemary (*Salvia rosmarinus* Spenn., Lamiaceae).

**Figure 2.**
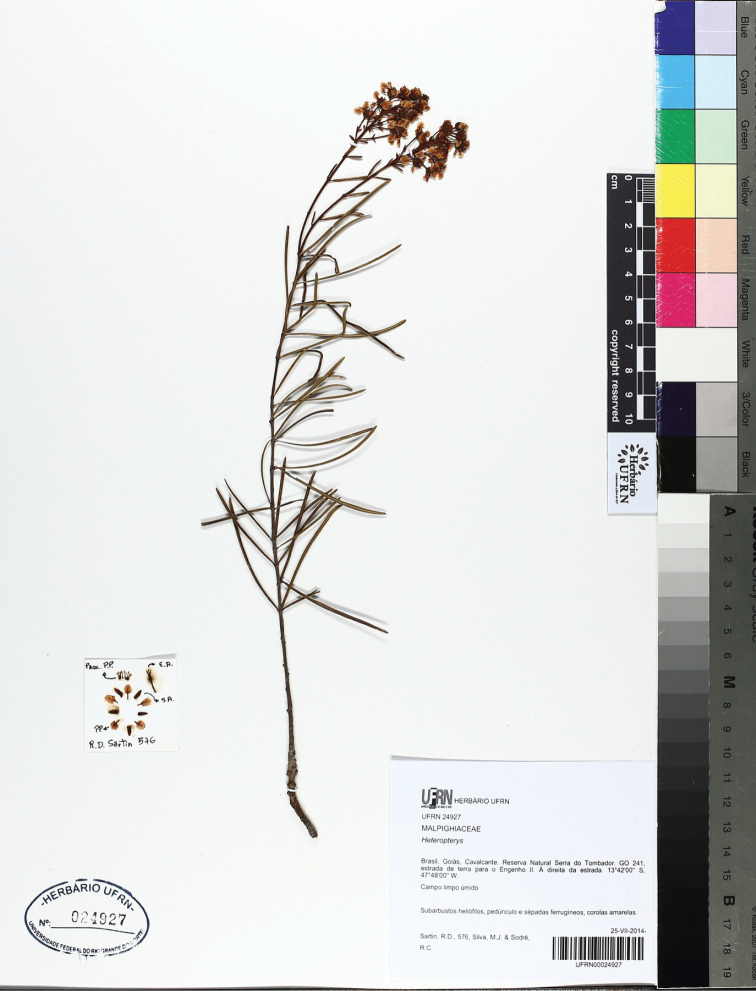
Holotype of *Heteropterys rosmarinifolia* R.F.Almeida & M.Pell. (R. Sartin et al. 576, UFRN barcode 00024927).

#### Notes.

*Heteropterys rosmarinifolia* is easily distinguished from the remaining species of the Parabanisteria group by the vegetative characters presented in the abovementioned diagnosis. It is most similar to *H. pannosa* Griseb. due to its small stature, delicate branches, and narrow leaf-blades. However, it can be differentiated by its stems distally unbranched (vs. branched in the upper half in *H. pannosa*), leaf-blades linear to very narrowly oblong, strongly conduplicate, ascending to upright, and abaxially rusty-sericeous (vs. narrowly elliptic, conduplicate, patent, and abaxially greyish-lanate-tomentose), leaves associated with the inflorescence verticillate (vs. opposite to ternate), cincinni verticillate (vs. subopposite to opposite), and anthers glabrous with connectives white (vs. pubescent and brown).

**Figure 3.**
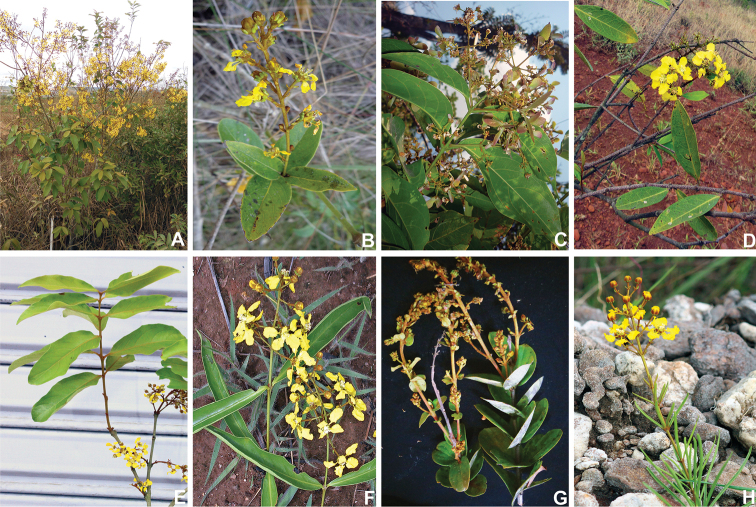
*Heteropterys* from the Parabanisteria group from the State of Goiás, Brazil **A***H. byrsonimifolia***B***H. coriacea***C***H. eglandulosa***D***H. escalloniifolia***E***H. nervosa***F***H. pannosa***G***H. procoriacea***H***H. rosmarinifolia*. **A** by L.C. Marinho **B, G** by I. Morais **C, H** by R. Sartin **D, E** by R.F. Almeida **F** by R. Ripley.

Despite fruits still being unknown for *H. rosmarinifolia*, they most likely are similar to the ones of *H. pannosa*, with the dorsal wing reduced to a crest, and nuts lacking lateral winglets (i.e., wingless nut). Alternatively, most species of the Parabanisteria group present a developed dorsal wing (Almeida et al. 2021). Floral and fruit characters are highly conserved in the Parabanisteria group and have been traditionally considered of little taxonomic relevance (Niedenzu 1928). Consequently, this group’s taxonomy currently heavily relies on vegetative characters related to life form, branch, and leaf morphology, rendering it the most taxonomic convoluted in *Heteropterys* (Niedenzu 1928; Almeida pers. observ.). Further studies are still necessary to properly explore the relevance of floral and fruit characters in this group and review species boundaries. For the time being, we provide below an identification key for the species of *Heteropterys* from the Parabanisteria group from the State of Goiás, Brazil.

### Key to the species of the Parabanisteria group from Goiás

Modified from Pessoa et al. 2014.

**Table:** 

1	Leaf-blades narrowly elliptic or linear to very narrowly oblong; thyrsi solitary	**2**
–	Leaf-blades elliptic, ovate-lanceolate, ovate or oblanceolate; thyrsi arranged in thyrsi	**3**
2	Leaf-blades narrowly elliptic, conduplicate, patent, abaxially greyish-lanate-tomentose, the ones associated with the inflorescence ternate; cincinni subopposite to opposite; anthers pubescent, connectives brown	***H. pannosa* (Fig. 3F)**
–	Leaf-blades linear to very narrowly oblong, strongly conduplicate, ascending to upright, abaxially rusty-sericeous, the ones associated with the inflorescence verticillate; cincinni verticillate; anthers glabrous, connectives white	***H. rosmarinifolia* (Fig. 3H)**
3	Lenticels lighter than the branch; leaf-blades abaxially sericeous to glabrescent	**4**
–	Lenticels darker than the branch; leaf-blades abaxially glabrous	**5**
4	Treelets; leaf-blades ovate, base obtuse, rounded or subcordate, margins plane; anterior style the same length as the posterior ones, anterior stigma pointing towards the center of the flower, the posterior ones pointing towards the latero-posterior petals	***H. byrsonimifolia* (Fig. 3A)**
–	Lianas; leaf-blades elliptic to oblanceolate, base cuneate, rarely obtuse, margins revolute; anterior style longer than the posterior ones, all stigmas pointing towards the posterior petal	***H. escalloniifolia* (Fig. 3D)**
5	Petioles sparsely tomentose to tomentose, sometimes glabrescent at age; anthers pubescent	***H. coriacea* (Fig. 3B)**
–	Petioles sparsely sericeous (glabrescent at age) or sericeous; anthers glabrous	6
6	Leaf-blades lanceolate to ovate-lanceolate; inflorescences axillary; styles with truncate apex	***H. nervosa* (Fig. 3E)**
–	Leaf-blades elliptic or ovate; inflorescences terminal; styles with uncinate apex	**7**
7	Lianas; leaf-blades elliptic, base cuneate; sepals eglandular, styles with apex curved-uncinate	***H. eglandulosa* (Fig. 3C)**
–	Subshrubs; leaf-blades ovate, base subcordate; sepals glandular, styles with apex straight-uncinate	***H. procoriacea* (Fig. 3G)**

## Supplementary Material

XML Treatment for Heteropterys rosmarinifolia
